# Congenital Zika Syndrome: Genetic Avenues for Diagnosis and Therapy, Possible Management and Long-Term Outcomes

**DOI:** 10.3390/jcm11051351

**Published:** 2022-03-01

**Authors:** Giuseppe Gullo, Marco Scaglione, Gaspare Cucinella, Arianna Riva, Davide Coldebella, Anna Franca Cavaliere, Fabrizio Signore, Giovanni Buzzaccarini, Giulia Spagnol, Antonio Simone Laganà, Marco Noventa, Simona Zaami

**Affiliations:** 1 Department of Obstetrics and Gynecology, Villa Sofia Cervello Hospital IVF UNIT, University of Palermo, 90146 Palermo, Italy; gullogiuseppe@libero.it (G.G.); gaspare.cucinella1@gmail.com (G.C.); 2 School of Medicine and Surgery, University of Palermo, 90127 Palermo, Italy; marco.scaglio95@gmail.com; 3 Department of Biomedicine, Neurosciences and Advanced Diagnostics, University of Palermo, 90133 Palermo, Italy; 4 Department of Women’s and Children’s Health, Padova Hospital, 35128 Padova, Italy; harianna.riva@gmail.com; 5 Department of Women’s and Children’s Health, Gynaecological Clinic, University of Padova, 35128 Padova, Italy; davide.colde@gmail.com (D.C.); giovanni.buzzaccarini@gmail.com (G.B.); giuliaspagnol.ts@gmail.com (G.S.); marco.noventa@gmail.com (M.N.); 6 Department of Gynecology and Obstetrics, Santo Stefano Hospital, ULS Toscana Centro, 59100 Rome, Italy; annafranca.cavaliere@uslcentro.toscana.it; 7 Department of Gynecology and Obstetrics, Sant’Eugenio Hospital, 00144 Rome, Italy; fabrizio.signore@aslroma2.it; 8 Department of Obstetrics and Gynecology, “Filippo Del Ponte” Hospital, University of Insubria, 21100 Varese, Italy; antoniosimone.lagana@uninsubria.it; 9 Department of Anatomical, Histological, Forensic and Orthopedic Sciences, “Sapienza” University of Rome, 00161 Rome, Italy

**Keywords:** Zika virus, pregnancy, microcephaly, prenatal diagnosis, neuroimaging, long-term outcomes, single nucleotide polymorphisms (SNPs)

## Abstract

Zika virus (ZIKV) was discovered in Uganda in 1947 and was originally isolated only in Africa and Asia. After a spike of microcephaly cases in Brazil, research has closely focused on different aspects of congenital ZIKV infection. In this review, we evaluated many aspects of the disease in order to build its natural history, with a focus on the long-term clinical and neuro-radiological outcomes in children. The authors have conducted a wide-ranging search spanning the 2012–2021 period from databases PubMed, PubMed Central, Web of Science, Medline, Scopus. Different sections reflect different points of congenital ZIKV infection syndrome: pathogenesis, prenatal diagnosis, clinical signs, neuroimaging and long-term developmental outcomes. It emerged that pathogenesis has not been fully clarified and that the clinical signs are not only limited to microcephaly. Given the current absence of treatments, we proposed schemes to optimize diagnostic protocols in endemic countries. It is essential to know the key aspects of this disease to guarantee early diagnosis, even in less severe cases, and an adequate management of the main chronic problems. Considering the relatively recent discovery of this congenital infectious syndrome, further studies and updated long-term follow-up are needed to further improve management strategies for this disease.

## 1. Introduction

ZIKV is a single-stranded ribonucleic acid (RNA) flavivirus transmitted by arthropod vectors, in particular, Aedes aegypti (or yellow fever mosquito, also responsible for the transmission of Dengue and Chikungunya) and Aedes albopictus (or Asian tiger mosquito, also responsible for the transmission of Chikungunya and West Nile virus). The virus replicates in the insect’s epithelial cells and, eventually, in the salivary glands. ZIKV infection used to be considered a tropical disease, but global interest has increased since 2015 due to an epidemic of neonatal microcephaly in Brazil. The virus has been found capable of crossing the placenta in all gestational periods, but mainly in the first trimester. The infection has also been shown to be transmissible sexually and through transfusions [1]. Another important aspect is that the vector Aedes aegypti varies seasonally in the United States [2]. No treatment or vaccine as yet exists for ZIKV. Symptomatic infection is characterized by low-grade fever, maculopapular rash, arthralgia, and conjunctivitis (some cases of Guillain-Barré syndrome have also been reported in adults); although the symptoms may last for about 5–7 days, 80% of cases are asymptomatic, which greatly complicates the diagnosis in pregnancy. Congenital ZIKV infection may accurately be viewed as a newcomer of the TORCH complex, an acronym that stands for toxoplasmosis, others (syphilis, varicella-zoster, parvovirus B19), rubella, cytomegalovirus (CMV), herpesvirus (HSV), given the characteristics it shares with such well-known pregnancy diseases with mild symptoms in the mother, vertical transmission, severe anomalies in the new-born and maternal therapy, which often fails to improve the prognosis [3]. Although it is a relatively new disease, many national and international associations have already drawn up guidelines for ZIKV infection in pregnancy [4], stressing the need for wide-ranging interventions via family-based support programmes aimed at addressing the special needs of children with CZS neurodevelopmental disability in low- and middle-income countries [5,6], as well as the socioeconomic struggles and psychological impact burdening their families [7,8]. All such issues have been further worsened by the COVID-19 pandemic. As the SARS-CoV-2 curve of infections grew at the height of the pandemic, so did the strain caused by ZIKV on public health facilities in countries where CZS already posed a threat long before the current pandemic [9,10]. The two diseases do bear similarities in terms of clinical manifestations, particularly in the early stages; such an ambiguity can cause delays in diagnosis and treatment interventions, thus, fostering the spread of infection and raising the risk of adverse clinical outcomes. Delays in diagnosis and treatment due to symptoms similar to COVID-19 and other infectious diseases such as Dengue and typhoid fever have also been reported. In India and Pakistan [11,12], the similarity between COVID-19 symptoms and other diseases has reportedly caused misdiagnosis and possible underreporting. To make matters worse, a diversion of resources away from entomological control services, in order to tackle COVID-19, can cause a higher rate of infections from other arboviruses (primarily Dengue and Chikungunya) spread by the same vector [13].

## 2. Materials and Methods

The authors have conducted a wide-ranging search spanning the 2012–2021 period from databases PubMed, Web of Science, Medline, and Scopus using search strings consisting of keywords such as Zika, pathogenesis, epigenetics, and microcephaly. Among these papers, we found review/meta-analysis, cohort/retrospective cohort studies (focused on long-term follow-up of children’s health) and case-report/case series (reporting the autopsy findings of fetuses that died in utero or shortly after birth). The results and evidence found in the various studies were schematized and both points of agreement and divergence were discussed. The various sections are designed to comprehensively describe the ZIKV congenital infection syndrome. This schematization was used to discuss and propose possible management strategies.

## 3. Pathogenesis

ZIKV has a unique ability among flaviviruses to cross the placental barrier (for example, Dengue virus does not have the same capacity) [14]. Hypothesized transmission routes are: direct infection of the syncytiotrophoblast layer, passage through ruptures in the syncytiotrophoblast layer, crossing of the syncytiotrophoblast layer through non-replicative routes (for example antibody-mediated transcytosis), infection of extravillous trophoblast or other more permissive placental cells (for example, cell of the maternal microvasculature and/or decidua), transmission to the fetus and/or villous core through maternal cells (most probably of immune origin), ascending vaginal infection [15]. The placental damage caused by the virus is related to local inflammation and/or metabolic alterations with mitochondrial dysfunction and loss of function of membrane lipids; the latter seem to rearrange themselves and to support viral replication [16]. ZIKV can reportedly upregulate the innate immune response of the decidua (an aspect in common with CMV), while some findings also show that the mechanism of damage of the maternal-fetal interface is not inflammation-mediated, but only related to the upregulation of apoptosis in placental cells [17]. Once the ZIKV has reached the fetal bloodstream, given its marked neurotropism, the damage is mainly neuronal. The pathogenetic mechanism has been hypothesized to be inflammatory initially for toxoplasma, CMV and rubella, the three most studied etiological agents that cause microcephaly by congenital infection [18]. Studies have also shown how the marked neurotropism of the virus may depend on a specific receptor on neural progenitor cells. In murine animal models, ZIKV infection has, at times, exhibited interference with cell cycle progression; neuronal cells may, therefore, become unable to migrate in the genesis of the telencephalon; for this reason, neurospheres and brain organoids cannot develop [19]. Reportedly, 1 to 42% of embryos or fetuses that are exposed to ZIKV infection in utero develop congenital Zika syndrome (CZS) [20]. Still, it is worth noting that the discovery of the viral teratogenic potential associated with ZIKV is relatively recent; hence, the degree of susceptibility and the mechanisms involved in the adverse effects on embryos and developing fetuses exposed to it are still not fully understood [21]. It is, therefore, essential to evaluate how susceptibility to ZIKV teratogenesis can be affected by environmental and genetic factors (e.g., the inflammatory process of response to ZIKV as risk or protective factors for CZS and the involvement of genetic variants in such dynamics).

## 4. Genetic Risk Factors

Technological advances are increasingly contributing to clarifying the key factors that affect and shape the dynamics of emerging infections, including ZIKV. Techniques such as genome-wide association studies [22], high-throughput sequencing [23] and screening based on clustered regularly interspaced short palindromic repeat (CRISPR) [24,25] has made it possible to identify human single nucleotide polymorphisms (SNPs), which may substantially affect the outcome of infectious diseases [26]. Polymorphisms in NOS2 and TNF genes have been reported to play a role in the development of CZS, with a higher risk in the first trimester, and of severe microcephaly. Specifically, genes closely linked to immune and inflammatory response, such as VEGFA, PTGS2, NOS3, TNF, and NOS2, with functional genetic variants, can exhibit different allelic, genotypic, and haplotypic frequencies in children with CZS [27]. A higher prevalence of the rs2297518 allele A in the NOS2 gene has also been observed in children with CZS. Significantly, the iNOS protein activity is somehow influenced by functional polymorphism NOS2 rs2297518. Enhanced protein activity and a higher level of nitric oxide (NO) are also presumably associated with A allele [28,29]. Neurogenesis and neurodevelopment have, in turn, been found to be substantially influenced by NO, whose dysregulation may impact the progression of various neurodevelopmental, neurobehavioral, and neurodegenerative disorders [30]. The rate of progression and development could be influenced by NO dysregulation linked to the individual response to ZIKV infection and by the patient’s genotype, even to the point of bringing about a congenital anomaly. Furthermore, CZS pathogenesis may be affected by single nucleotide variants: rs2076469 at the DISP3 gene and other rare variants in the IL12RB2 gene, due to their possible protective value and connection with neuronal proliferation and differentiation. Both phenotypes have been found altered in CZS patients, which may impact early immune-response to ZIKV infection [31,32,33]. Recent findings also reflect how two genes prominently involved in the regulation of bone development and cell–cell adhesion, FGFR3 and ITGB4, are upregulated in the brains of CZS patients [34].

## 5. Reverse Genetics and Recombinant ZIKV

The complex ways in which the pathology of ZIKV infection is affected by genetic traits will be instrumental in laying out therapeutic pathways and enhancing surveillance and prevention capabilities to counter the spread of ZIKV [35]. Reverse genetic strategies, either in vitro or in vivo, may constitute a valuable approach through which research will gain a more thorough understanding as to ZIKV’s biology and pathogenesis. The creation of recombinant viruses with specific mutations through the manipulation of the ZIKV genome has enabled researchers to shed a light on the function of viral proteins, the interactions involved between ZIKV and its host, and associated disease [36]. Research strategies harnessing reverse genetics have also proven valuable in the development of new effective prevention and control measures [37,38]. The bioengineering of replicating-competent recombinant ZIKV, which can be used to modify the ZIKV genomic RNA at the DNA level and express reporter genes, has, in fact, shown remarkable potential for the development of anti-viral therapeutic options to treat ZIKV infection [39,40], provided that the instability commonly linked to the construction of full-length cDNA clones can be overcome [41,42]. New research avenues are currently being pursued, relying on novel methods for more efficient and easier infectious clone construction, for instance, through the use of homologous recombination clones during plasmid construction [43].

## 6. Prenatal Diagnosis

The gold standard for prenatal diagnosis of ZIKV infection is the polymerase chain reaction essay (PCR) on amniotic fluid. Virus-specific IgM antibodies may be detected 3 days after the onset of symptoms. The serology findings can determine false positives due to cross-reaction with other flaviviruses (like West Nile virus, Dengue and yellow fever). After birth, the viral genome is detectable in saliva, breast milk, urine, and serum within several days after delivery; these data support the possible perinatal transmission of the infection [1].

Another limitation for prenatal diagnosis of the infection is persistent viremia and viruria in pregnancy, even weeks and months after primary exposure [44]. In the case of microcephaly, defined head circumference <2SDS is detectable by ultrasound starting from the second trimester, whereas ZIKV infection occurs typically in the first trimester. Other ultrasound signs suggestive of infection include ventriculomegaly, brain calcifications and posterior fossa destructive lesions; these findings are not found in other congenital infections [45]. According to some studies, another typical sign is disproportion in fetal growth, such as an unusual femur sparing growth restriction [46]. Indicators for diagnosis of ZIKV infection in the first trimester have been summarized in Table 1.

## 7. Clinical Signs

The distinctive features of the congenital ZIKV syndrome in the new-born are the following: severe microcephaly with partially collapsed skull, redundant scalp skin, early closure of the fontanelles, macular scarring and focal pigmentary retinal mottling, congenital contractures of one or more joints (similar to arthrogryposis, very rare in other congenital infections) and extrapyramidal motor symptoms with marked hypertonia. According to some authors, microcephaly may develop in new-borns with a normal head circumference at birth [1,47]. In severe cases, placental insufficiency caused by infection can lead to fetal growth restriction and, sometimes, even to fetal demise [6,48].

## 8. Neuroimaging

Neuroimaging with computed tomography (CT) or magnetic resonance (MRI) has found calcifications of the cortico-medullary junction with band distribution, more frequently in the basal ganglia and less commonly in the thalamus. Other alterations include cysts, mainly in the occipital area, delayed myelination and ventriculomegaly (Table 2). The latter, if severe, may require ventricular-peritoneal shunt surgery. Some of the alterations mentioned above may be present even in children with normal head circumference [1].

In congenital ZIKV infection, the whole development of the brain is compromised, with a reduction in cortical gyrification, cerebellar hypoplasia, and hypo/dysmyelination of the white matter in almost all affected subjects. These pathologic alterations are visible both with CT performed after birth and with fetal MRI [49].

Vasco Aragao et al. and Pires et al. have reported that the calcifications of the junction between the cortical area and the white matter are the most frequent finding, followed by alterations of the gyrification, such as pachygyria and micropolygyria, especially in the frontal lobes; hypoplasia of the corpus callosum, ventriculomegaly, hypoplasia of the cerebellum and brainstem (mainly of the pons) or global delay of myelination are less frequent [50,51]. Calcifications of the cortico-medullary junction, although described by most authors, should not be considered among the major criteria to define the prognosis since they tend to disappear with age in most children (92–95%). Therefore, this change is not accompanied by clinical improvement; the absence of calcifications does not exclude the diagnosis of the disease [52]. The pathological definition of the disease has proven particularly challenging, as mild and/or unspecific placental findings in ZIKV-affected pregnancies have been reported, e.g., chronic placentitis, increased Hofbauer cells, chronic villitis, variable perivillous fibrin and mononuclear cells, and villous immaturity, among others [53]. Assessing the risks and severity of ZIKV infection has proven challenging, mostly because of the dearth of specific diagnostic reagents and of a still incomplete understanding as to its pathogenic patterns, mechanisms and molecular virology [54,55].

Comparing MRI pathological changes of ZIKV infection with those of CMV infection, (Table 3), the spectrum of malformations appears to be very similar, yet more severe [56].

## 9. Long-Term Developmental Outcomes

The impact of congenital Zika virus infection on the brain of children has been investigated by several authors through autopsy findings in fetuses (stillbirths or interrupted pregnancy) and children who died shortly after birth. Mlakar et al., in a fetal autopsy, observed complete agyria, calcifications of the junction between the cortex and white matter, hydrocephalus and focal inflammation loci. These alterations are consistent with the neuroimaging described in the previous paragraph. Molecular biology investigations with RT-PCR on these tissues led to isolation of the viral genome [57].

Brasil Martines et al. analyzed brain tissue of children who died after birth with microcephaly and severe arthrogryposis and placental tissues of first trimester miscarriages. In both cases, it was possible to detect viral antigens and viral genome with RT-PCR technique [58].

In children with minor morphological damage, the main neurological problems are infantile cerebral palsy, epilepsy (the extensive damage caused by the Zika virus on the cortical structures of patients can lead to higher cumulative incidence of epilepsy episodes of higher severity, in addition to a worse response to anti-epileptic treatment [59]) and neurodevelopmental repercussions [60,61]. Wheeler et al., in a 30-months follow-up study of 121 children affected by congenital ZIKV infection, reported global developmental delay in almost all specific domains (in particular, cognition, fine motor skills and expressive language). According to these authors, the most indicative predictor of future developmental outcome is head circumference at birth [62]. In another study of 444 children with 12-month follow-up after birth, about 8 out of 10 children exposed to prenatal infection, but without microcephaly, were found to be healthy. Bilateral spastic infantile cerebral palsy has been reported in children with congenital microcephaly. The general movement assessment (GMA) at 3 months of life proved to be an accurate diagnostic test that should be introduced into the diagnostic routine [63]. Bertolli et al. carried out a 24-months follow-up of children with congenital infection ascertained by laboratory and ultrasound anthropometric criteria before birth (or only one of the two). Neurological and psychomotor outcomes of children with positivity for both criteria were worse, while less severe outcomes were reported in children with congenital infection ascertained solely by the laboratory criterion. An accurate monitoring of neurological development is necessary even when prenatal infection is uncertain [64]. Other issus observed in children affected by congenital ZIKV infection are failure to thrive, cardiac malformations, excess nuchal skin, auditory abnormalities and eye abnormalities. Even children with normal head circumference may develop neurological problems, such as hyperreflexia, abnormal tone, congenital neuromotor signs and feeding difficulties. In addition, over half of children with severe forms (54%) develop epilepsy [65,66]. Table 4 summarizes the possible clinical signs of congenital ZIKV syndrome. The spectrum of signs can vary widely; they are not always present concomitantly.

## 10. Conclusions

**Figure 1:**
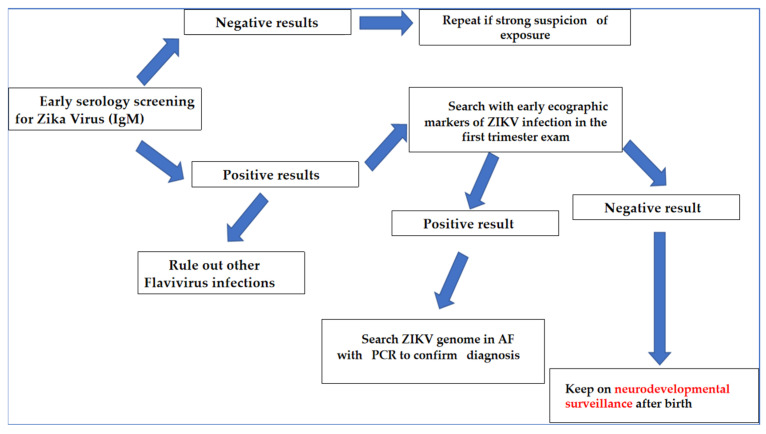
Algorithm of diagnosis for ZIKV infection in endemic countries.

This review set out to analyze the main features of congenital ZIKV syndrome, including the pathogenesis of placental and neuronal damage, the methods of prenatal diagnosis, the clinical diagnosis of affected children, neuroimaging and long-term developmental outcomes. Given the large percentage of asymptomatic infections in pregnant women, screening seems to be the only method that really guarantees an early diagnosis and serology, with IgM detection. It should, therefore, be proposed to all pregnant women in endemic areas. Since this test is easily burdened by false positives in the endemic areas, it should be associated with obstetric ultrasound to detect specific markers already in the first trimester of pregnancy. Everyone should be aware that microcephaly can only be seen in the second trimester ultrasound and that some of the affected children will not develop microcephaly. In the case of strong clinical suspicion, PCR on amniotic fluid ought to be carried out (Figure 1).

**Figure 2:**
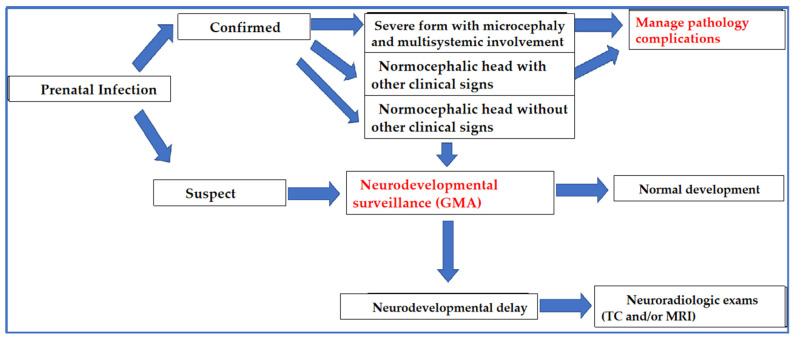
Algorithm of postnatal management of suspected congenital ZIKV infection.

Long-term outcomes of affected children are another very important point of interest. The majority of children who develop microcephaly have an overall developmental delay and, often, severe cerebral palsy. Conversely, among children with normal head circumference, most have a regular pattern of development. In these cases, with established prenatal exposure, even in the absence of microcephaly, it is important to monitor regular development with accurate tests such as GMA. Obviously, GMA, in addition to suggestive clinical signs, can also help to assess cases that have eluded prenatal diagnosis (or doubtful cases due to isolated laboratory positivity). Knowledge of the neuroradiological markers of infection is certainly helpful in cases of clinical suspicion. Children diagnosed with neurodevelopmental issues should start specific rehabilitation protocols as soon as possible. Furthermore, it is essential to investigate epilepsy, possibly with serial electroencephalographic insights and monitoring (Figure 2).

In conclusion, shedding light on key aspects of this disease will allow for early diagnosis, even in less severe cases, and adequate management of chronic issues. Given the relatively recent discovery of this congenital infection syndrome, further studies, with updated long-term follow-up of affected children are needed to further improve management strategies.

## Figures and Tables

**Table 1:** Indicators for diagnosis of ZIKV infection in the first trimester.

Diagnosis	Exam Timing	Morphological Features	Further Remarks
ZIKV infection	Polymerase Chain Reaction analysis of amniotic fluid best period to perform an amniocentesis is between the 21st and 22nd week		Around 6–9 weeks are required after maternal infection for the virus to be eliminated in the fetal urine in amounts detectable in the amniotic fluid
Anti-CMV IgM antibodies Virus-specific IgM antibodies may be detectable >3 days after onset of illness	Blood exam	No detectable virus-specific IgM antibodies in serum collected within 7 days of illness onset	IgM testing should be repeated on a convalescent-phase sample to rule out infection in the mother with a clinical syndrome suggestive of ZIKV infection. IgM antibodies are only present in 70% infected babies
Microcephaly	Neurosonographic approaches for the detection of malformations	Head circumference < 2SDS	An estimated 1% to 13% risk of microcephaly is associated with maternal infection in the first trimester of pregnancy
Ventriculomegaly	Ultrasound examination	Atrial diameter ≥ 10 mm on prenatal ultrasound	Roughly 5% of cases of mild to moderate ventriculomegaly reportedly arise from congenital fetal infections, such as CMV, toxoplasmosis and ZIKV
Brain calcifications Posterior fossa destruction lesions	Ultrasound examination; MRI	More visible in II-III trimester	Punctate calcifications between the cortex and subcortical white matter
Disproportion in fetal growth	Ultrasound	Femur-sparing profile of growth restriction	Infection in the first trimester is linked to the highest risk of structural and developmental anomalies
Germinolytic cysts (GLC) and lenticulostriate vasculopathy (LSV)	Transvaginal scan		Found in up to 37% of newborns exposed to ZIKV in utero, might constitute potential risk factors for worse early neurodevelopmental outcomes
Cerebellar hypoplasia and migrational disorders such as polymicrogyria (PMG)	MRI		Polymicrogyria and pachygyria mostly detected in the frontal lobes

**Table 2:** MRI findings in congenital ZIKV infection.

CT ^1^ and MRI ^2^ Findings in Congenital Zika Syndrome
Punctate calcifications (basal ganglia > thalami)
Severe ventriculomegaly
Global delayed or hypo-myelination
Pachygyria or polymicrogyria (mostly in the frontal lobes)
Hypoplasia of the cerebellum and the brainstem.
Enlarged cisterna magna
Abnormalities of corpus callosum (hypoplasia/hypogenesis)
Cysts/Pseudocysts (mainly in the occipital area)

^1^ CT: computed tomography; ^2^ MRI: magnetic resonance.

**Table 3:** MRI findings in congenital CMV infection.

MRI Findings in Congenital CMV ^1^ Infection
White matter hyperintensities
Ventriculomegaly
Ventriculitis
Calcifications
Cysts/Pseudocysts

^1^ CMV: Cytomegalovirus.

**Table 4:** Clinical signs of congenital ZIKV syndrome.

Congenital Zika Syndrome Clinical Signs
Microcephaly
Hydrocephalus
Cerebral Palsy
Epilepsy
Neurodevelopmental disorders
Ear and Eye abnormalities
Cardiac malformations
Growth delay

## Data Availability

Upon request.
